# Effect of Nicotine on CYP2B1 Expression in a Glioma Animal Model and Analysis of CYP2B6 Expression in Pediatric Gliomas

**DOI:** 10.3390/ijms19061790

**Published:** 2018-06-16

**Authors:** Sonia Nava-Salazar, Saúl Gómez-Manzo, Jaime Marcial-Quino, Alfonso Marhx-Bracho, Bryan V. Phillips-Farfán, Carlos Diaz-Avalos, America Vanoye-Carlo

**Affiliations:** 1 Departamento de Inmunobioquímica, Instituto Nacional de Perinatología IER, Secretaría de Salud, 11000 Ciudad de México, Mexico; s_nava_s@yahoo.com.mx; 2 Laboratorio de Bioquímica-Genética, Instituto Nacional de Pediatría, Secretaría de Salud, 04530 Ciudad de México, Mexico; saulmanzo@ciencias.unam.mx; 3 CONACyT—Instituto Nacional de Pediatría, Secretaría de Salud, 04530 Ciudad de México, Mexico; jmarcialq@ciencias.unam.mx; 4 Departamento de Neurocirugía, Instituto Nacional de Pediatría, Secretaría de Salud, 04530 Ciudad de México, Mexico; marhxalfons@yahoo.com.mx; 5 Laboratorio de Nutrición Experimental, Instituto Nacional de Pediatría, Secretaría de Salud, 04530 Ciudad de México, Mexico; bvphillips@gmail.com; 6 Departamento de Probabilidad y Estadística, Instituti de Investigaciones en Matemáticas Aplicadas y en Sistemas, UNAM, 04510 Ciudad de México, Mexico; carlos@sigma.iimas.unam.mx; 7 Laboratorio de Neurociencias, Instituto Nacional de Pediatría, Secretaría de Salud, 04530 Ciudad de México, Mexico

**Keywords:** CYP2B6, CYP2B1, glioma, cyclophosphamide, pediatric, brain

## Abstract

Cyclophosphamide (CPA) is a pro-drug commonly used in the chemotherapeutic schemes for glioma treatment but has high toxicity and the side effects include brain damage and even death. Since CPA is activated mainly by CY2B6, over-expression of the enzyme in the tumor cells has been proposed to enhance CPA activation. In this study, we explored the induction of the *Cyp2b1* (homologous to *CYP2B6*) by nicotine in an animal rat model with glioma. Gene expression and protein levels were analyzed by RT-PCR and Western blot. Nicotine treatment increased CYP2B1 protein levels in the healthy animals’ brain tissue. In the brain tissue of animals with glioma, the CYP2B1 showed a high expression, even before nicotine treatment. Nicotine did not increase significantly the CYP2B1 protein expression in the tumor, but increased its expression in the tumor vicinity, especially around blood vessels in the cortex. We also explored CY2B6 expression in glioma samples derived from pediatric patients. Tumor tissue showed a variable expression of the enzyme, which could depend on the tumor malignancy grade. Induction of the CYP2B6 in pediatric gliomas with lower expression of the enzyme, could be an alternative to improve the antitumoral effect of CPA treatment.

## 1. Introduction

In children, central nervous system tumors (CNST) are the main cause of death due to cancer and represent the second most common type of cancer after leukemia [1,2,3]. Gliomas are the most common central nervous system neoplasm and are derived primarily from glial progenitor cells [4,5]. The majority of childhood gliomas are low-grade tumors (WHO Grade I–II); however, high-grade gliomas (WHO-III–IV) account for approximately 8–12% of all CNST cases [3,5,6]. Surgical resection is the first treatment alternative, however when surgery is not feasible due to tumor location or progress, chemotherapy is the most common treatment option for pediatric gliomas [1,2,5].

Oxazaphosphinans are an important group of therapeutic agents due to their anti-tumor and immunosuppressive properties. These drugs include cyclophosphamide (CPA), which is a secondary line alkylating chemotherapeutic agent used in combination with other agents, for a wide range of malignancies. It is administered by oral or parenteral routes in a wide range of doses. CPA is a therapeutically inactive pro-drug and its bio-activation and degradation have been extensively studied in various species. In rats and humans, the liver is the main organ that participates in CPA bio-activation. Approximately 70–80% of the administered dose is metabolized by enzymes of the cytochrome P450 (CYP450) family to hydroxylated metabolites [7,8,9].

**Figure 1:**
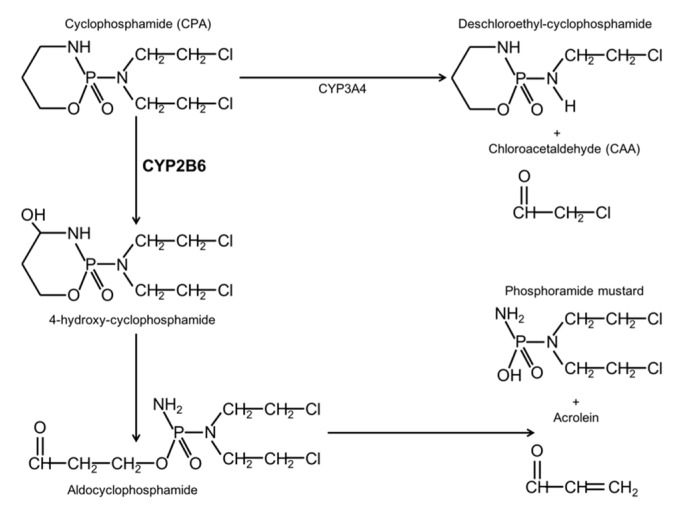
Role of CYP2B6 in cyclophosphamide metabolism. CPA prodrug is mainly activated by CYP2B6 by hydroxylation. The 4-hydroxy-CPA is decomposed to phosphoramide mustard, the DNA alkylating metabolite with antitumoral activity. CYP3A4 is the main enzyme responsible of the dechloroethylation of CPA and CAA formation, which have severe toxic effects.

CPA is metabolized by constitutive P450 enzymes belonging to the CYP2C subfamily as well as by the drug-inducible enzymes CYP2B and CYP3A. CYP2B1 is the most active catalyst of CPA in the rat liver, whereas the corresponding human enzyme is CYP2B6. CYP2B1 is responsible for about 45% of CPA hydroxylation, while CYP3A4 and CYP2C9 contribute to around 25% and 12%, respectively. CPA hydroxylation generates 4-OH-cyclophosphamide, an unstable compound that forms the phosphamide mustard, which is responsible for the antitumoral effect by DNA alkylation in proliferating cells. On the other hand, the lateral chains of CPA can be oxidized, this reaction is catalyzed mainly by CYP3A4 and it forms chloroacetaldehyde (CAA). CAA has neurotoxic, nefrotoxic, cardiotoxic, and myelosuppressive properties [5,6,7,8] (Figure 1).

During the nineties, there was growing interest in using chemotherapeutic schemes with high doses of CPA in glioma chemotherapy, with the objective of augmenting the clinical response. Treatment of highly invasive or recurrent pediatric gliomas with medium to high doses of CPA results in higher disease-free survival compared to a low dose regimen. However, the use of CPA at high doses also increases the toxicity caused by this compound [7,8,10,11,12,13]. More recently, CPA use for high grade glioma treatment and recurrent tumors has regained interest and new strategies to diminish its side effects have been proposed [14,15,16,17,18].

Since CYP2B6 has high hydroxylase activity for CPA, it has been proposed to sensitize tumor cells to the action of this drug by in situ expression of this enzyme with gene therapy. This would improve therapeutic efficiency by increasing tumor cell death with a lower drug dose and thus considerably reduce its toxicity [8,19,20]. Moreover, in 2013, Chiocca and co-workers reported the toxicological and biodistribution data for the MGH2.1 oncolytic virus in animals, which expressed CYP2B1 and CPT11-activating secreted human intestinal carboxylesterase (shiCE). Modulating the activity of CYP450 enzymes could be an adequate strategy to improve the efficiency of CPA and similar drugs in the treatment of gliomas [7,8,9,15].

The CYP2B6 enzymes are induced by drugs, environmental toxins, and endogenous substances. Compounds, such as phenobarbital, are the most potent CYP2B6 inducers. However, these compounds also act upon the CYP3A family and their action are not tissue-specific [21,22]. CYP2B6 is expressed primarily in the liver and it has been identified in several areas of the human brain. It is located in neurons and glial cells, mainly astrocytes surrounding blood vessels. An important increase in its expression was found in brain tissue from smokers; suggesting that this enzyme can be induced by nicotine [22]. In fact, chronic nicotine treatment induces *CYP2B6* expression (91% homology with the human gene) in the brain of African green monkeys. This occurred in several cell types, among them, pyramidal cells of the frontal cortex, Purkinje cells of the cerebellum and neurons of the substantia nigra; however, its expression in the liver was not affected [23]. In a similar manner, nicotine treatment induced the expression of the *Cyp2b1* gene (*CYP2B6* homologous) in several cell types of the rat brain, mainly in the frontal cortex [24].

Using an animal model of glioma, we characterized the changes in the *Cyp2b1* expression after the administration of nicotine, in brain tissue of heathy animals as well as in the glioma. Moreover, CYP2B6 expression was explored in samples of pediatric gliomas, ranging from grade I to IV. We found that the expression of this enzyme is high in the glioma tissue and differs between samples, probably according to malignancy grade.

## 2. Results

### 2.1. Nicotine Did Not Affect Cyp2b1 Gene Expression Levels

**Figure 2:**
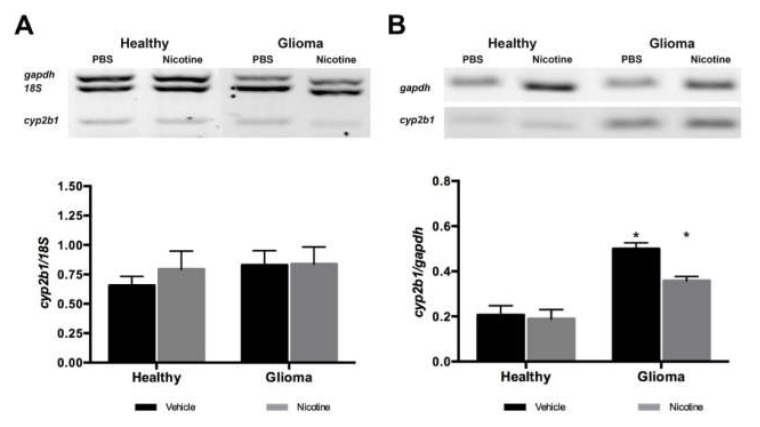
Effect of nicotine on *Cyp2b1* mRNA levels in the liver and brain. *Cyp2b1* mRNA levels were analyzed by multiplex RT-PCR. (**A**) Representative RT-PCR multiplex for *Cyp2b1* detection in liver tissue. Nicotine treatment did not modify *Cyp2b1* mRNA in the liver of healthy rats or animals with glioma; (**B**) Representative RT-PCR multiplex for *Cyp2b1* detection in brain tissue. Glioma brain tissue expressed higher levels of *Cyp2b1* mRNA compared to normal brain tissue and nicotine treatment do not change the *Cyp2b1* gene expression (* *p* < 0.05 vs. healthy vehicle). Data represent the means ± SE. *18S* and *Gapdh* were used as reference controls.

Since it has been reported that nicotine induces *Cyp2b1* expression in the brain but does not affect liver expression, we analyzed *Cyp2b1* mRNA levels in a murine glioma model treated with nicotine. *Cyp2b1* mRNA expression in the liver of healthy animals, treated with vehicle or nicotine, showed no statistical differences. Animals that developed glioma tumors showed similar levels of *Cyp2b1* expression in the liver as healthy animals without statistical differences (Figure 2a). Brain *Cyp2b1* expression was also analyzed; nicotine treatment did not change *Cyp2b1* mRNA levels in healthy rats. Unexpectedly, brain tissue of animals with glioma, showed a higher level of *Cyp2b1* expression when compared with healthy animals, and after nicotine treatment, the *Cyp2b1* expression did not increase (Figure 2b). This data suggests a change in the *Cyp2b1* regulation when a glioma tumor has developed.

### 2.2. Brain Tissue with Glioma Express High Levels of CYP2B1 Protein

**Figure 3:**
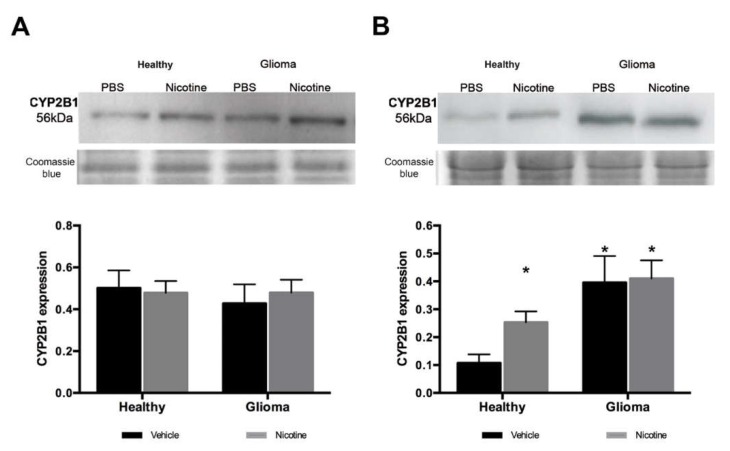
CYP2B1 protein expression in the liver and brain. CYP2B1 protein levels were analyzed by Western blot. (**A**) CYP2B1 protein detection in liver tissue. Densitometric analysis showed that nicotine treatment did not modify CYP2B1 expression in the liver; (**B**) CYP2B1 protein detection brain tissue. CYP2B1 protein levels in the brain were analyzed by densitometry. Nicotine treatment induced the enzyme protein in healthy animals. The tumor tissue expressed higher levels of CYP2B1 protein and nicotine treatment did not change it significantly (* *p* < 0.05 vs. healthy vehicle). Coomassie blue gel staining is presented to verify protein load. Data represent the means ± SE. * *p* < 0.05.

Nicotine treatment for seven days did not modify CYP2B1 protein expression in the liver of healthy animals or in rats with glioma (Figure 3a). While nicotine treatment in healthy animals increased CYP2B1 protein by almost 2.5 (*p* < 0.05) times compared with brain tissue of animals receiving vehicle (Figure 3b). Western blot analysis revealed high variability of CYP2B1 expression in brain tissue corresponding to the tumor hemisphere, which could be due to the variability in tumor size. Clearly, an overexpression of CYP2B1 was detected even without nicotine treatment (Figure 3b) and nicotine administration did not significantly affect CYP2B1 expression in brain tumor tissue. However, CYP2B1 expression was slightly increased compared to vehicle in the tumor tissue after nicotine treatment (Figure 3b).

### 2.3. Nicotine Treatment Modified CYP2B1 Distribution in Tumor Brain Tissue

**Figure 4:**
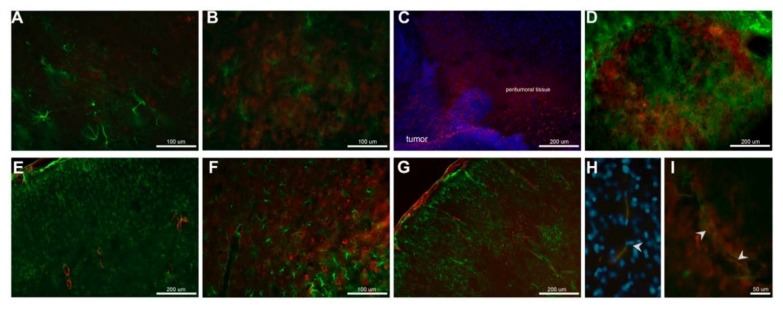
Brain CYP2B1 protein localization. Double immunofluorescence for CYP2B1 (red) and GFAP (green) was performed (**A**–**G**). CYP2B1 was mainly expressed in neuronal cells in healthy brain tissue (**A**) and nicotine treatment increased its expression (**B**). CYP2B1 was highly expressed in tumor cells and peritumoral tissue**,** nuclei were counterstained with DAPI (**C**). High detection of the enzyme was found in the glioma and not in cells expressing GFAP (**D**), the tissue away from the tumor did not express CYP2B1 (**E**). Nicotine treatment did not increase CYP2B1 expression in tumor cells (**F**); however, it increased CYP2B1 expression in the vicinity of the tumor, along blood vessels (**G**). Finally, blood vessels were identified with von Willebrand protein (green) and its co-location (yellow) with CYP2B1 is shown (arrows) in normal (**H**) and tumor vessels (**I**).

In order to explore the distribution of CYP2B1 in the brain tissue of animals with glioma, we performed an immunofluorescence for the CYP2B1 protein (red) and glial fibrillary acidic protein (GFAP; green) (Figure 4). Basal CYP2B1 expression in healthy animals was present mainly in neuronal (Figure 4a). Nicotine treatment increased CYP2B1 expression in the cortex (Figure 4b).

In the brain tissue of animals with glioma, a very high expression of the enzyme was localized in the peritumoral tissue (Figure 4c) and tumor cells (Figure 4c,d) and a lower expression in the tumor vicinity (Figure 4e). After nicotine treatment, expression of CYP2B1 in tumor cells remained unaffected (Figure 4f). However, the enzyme expression increased in the vicinity of the tumor, especially in structures that resemble blood vessels of the cortex (Figure 4g). A double immunofluorescence confirmed the co-expression of CYP2B1 near and in blood vessels, both in normal (Figure 4h) and tumor vessels (Figure 4i). These data confirm that the main source of CYP2B1 protein detected by Western blot in the brain tissue of glioma animals was the tumor itself. Moreover, although nicotine treatment did not modify CYP2B1 levels in the glioma, the peritumoral tissue presented a response very similar to the brain tissue of healthy animals treated with nicotine.

### 2.4. CYP2B6 Expression in Human Brain Gliomas

**Figure 5:**
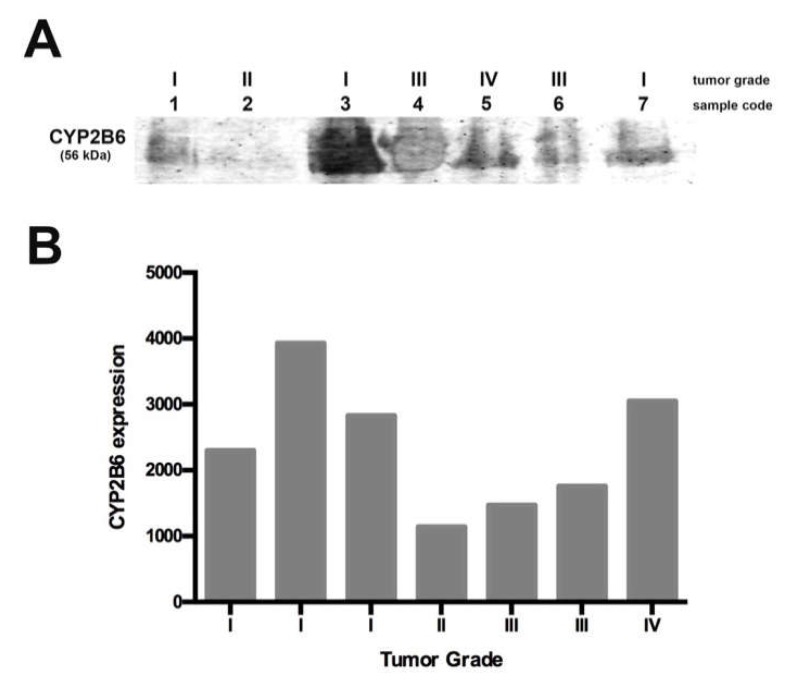
CYP2B6 protein expression in human gliomas. (**A**) Expression of CYP2B6 in pediatric samples with different grade gliomas; (**B**) Densitometric analysis (arbitrary units) of CYP2B6 expression according to tumor grade.

Since we found CYP2B1 over-expression in the animal glioma model, we explored the expression of the homologous enzyme (CYP2B6) in samples from human gliomas. We focused on pediatric glioma because it is the most common pediatric brain tumor, representing the major cause of morbi-mortality in children. We explored CYP2B6 expression in seven glioma samples, five samples were obtained from male patients and two from females, with an age range between 3–14 years and pathological diagnoses of glioma grade I-IV (Table 1). Using Western blot, we found differences in enzyme expression in samples of low vs. high-grade gliomas (Figure 5a). We found the lowest expression in grade II and III tumors and the highest in grade I and IV tumors. Note that the first sample, corresponding to a grade I tumor, was the only one obtained from a recurrent tumor. Before surgery, the patient received vincristine and carboplatin-based chemotherapy as well as radiotherapy (64 G). However, is necessary explore the enzyme expression in a greater number of samples (Figure 5b).

## 3. Discussion

Cyclophosphamide is a highly effective antitumoral agent used in combination with other chemotherapeutic drugs, for the treatment of pediatric gliomas, however it is generally used as a second line drug due to its high toxicity. Several side effects have been associated to CPA use such as nephrotoxicity, gonadotoxicity, and possible cardiotoxic effects. In order to reduce CPA toxicity, different chemotherapy schemes and strategies have been proposed, as well as adjuvants [16,17,25]. Among these strategies, the sensitization of cancer cells to CPA antitumor effect by increasing the expression of enzymes related to its activation [7,8,9,15] has been addressed. In this line, we characterized CYP2B1 induction by nicotine, the main enzyme responsible from CPA activation, in a glioma animal model. We found high basal expression of the enzyme in tumor cells; thus, we also explored the expression of CYP2B6 in pediatric glioma samples.

Nicotine has been reported to specifically induce CYP2B6 in human brain tissue (CYP2B1 in the rat). As expected, in our model, nicotine treatment did not modify *Cyp2b1* (homologous to *CYP2B6* in the rat) gene expression in the liver of healthy or glioma animals. *Cyp2b1* gene expression in the brain of healthy animals remained unaltered. However, we found an important increase of gene expression in the brain in the presence of a glioma. These observations suggest that nicotine did not act as an inductor of CYP2B1 enzyme at the gene level and that the tumor itself expressed higher levels of the enzyme by modulating its expression at the transcriptional level. In agreement with previous reports, nicotine treatment increased *Cyp2b1* expression in healthy brain tissue at the protein level but not in the liver, showing that nicotine specifically induces this enzyme in the brain. The mechanism of enzyme induction by nicotine has not been described, but our results suggest that it occurs at the protein, not the gene, level.

In glioma brain tissue, the basal expression of the enzyme was higher compared to healthy brain tissue and nicotine treatment did not significantly affect CYP2B1 protein expression, but a slight increase was observed. Using immunofluorescence detection, we were able to visualize in situ CYP2B1 protein basal expression in tumor cells. Nicotine treatment increased its expression in peri-tumor tissue, especially in the brain cortex and near blood vessels.

Based on our results and in the main role of CYP2B1 enzyme in CPA bioactivation, we consider that the increased protein in the tumor vicinity, especially near blood vessels and cells near the tumor, could result in a better pro-drug activation, having a positive impact in glioma treatment with CPA. However, detailed studies exploring CPA tissue-specific kinetics and dynamics are needed in order to check if nicotine treatment previous to CPA based chemotherapy could enhance it antitumoral effect.

Since we found high tumor CYP2B1 protein expression in the animal model, we explored CYP2B6 protein levels in pediatric gliomas. We used seven samples collected for ten months, with tumor grades ranging from I to IV. We found that the enzyme was expressed in all the samples but at different levels; grade I and IV samples showed a higher enzyme expression, while grade II and III samples showed a lower expression. The differential expression of this enzyme in pediatric gliomas could be related to tumor malignancy and probably related to CPA response chemotherapy. In this study, we included a sample obtained from a recurrent grade I tumor, which showed lower expression of the enzyme when compared with the other grade I samples, more similar to grade II and III tumors. This observation could be related to the higher CPA doses required for treatment, as reported by Allen in 1981 [10] for recurrent gliomas. In this sense, it is important to explore if the use of a specific CYP2B6 inducer, such as nicotine, could improve CPA-based chemotherapy. Due to the low number of pediatric glioma samples analyzed in this study, a more detailed study must be conducted in order to elucidate if CYP2B6 induction as a pre-treatment could result in a better response to CPA.

Moreover, since chemo-resistance due to changes in the expression and activity of CYP450 enzymes has been widely documented in several cancer types [26], CYP2B6 tumor expression could be related to tumor therapy response. The presented results could also be relevant in order to choose the best chemotherapeutic option for pediatric gliomas treatment, however, it is necessary to perform more studies to know if CYP2B6 expression could be used as a CPA-based chemotherapy response marker. Although our results are not conclusive due to the sample number analyzed, it sets a precedent that requires follow-up.

Our results suggest that it is necessary to explore the CYP2B6 induction by nicotine in pediatric glioma tumors. This could be used to improve the response of gliomas to CPA based chemotherapy according to the tumor grade, without needing molecular sensitization of tumor cells and thus avoiding its risks. The animal model reported in this work could be used to explore changes in CPA pharmacokinetics and anti-tumor activity when pre-treatment to induce CYP2B6 is used, since the model has high basal expression of CYP2B6 in the tumor tissue as occurs in pediatric gliomas.

## 4. Materials and Methods

### 4.1. Animals

The experiments were conducted in accordance with the principles and procedures of the National Institutes of Health Guide for the Care and Use of Laboratory (SAGARPA NOM-062-Z00-1999). Male Fischer 344 rats, 8–10 weeks old (180–200 g), were housed 4/5 per cage at a temperature of 23 ± 3 °C, with 50 ± 10% relative humidity and 12:12-h light:dark cycle. Food pellets and water were available ad libitum.

### 4.2. Cell Culture

The rat RG2 glioma cell line a highly malignant cell line equivalent to tumor grade IV was cultured in Dulbecco’s modified Eagles’s medium-nutrient mixture F-12 (DMEM-F12, Thermo Fisher Scientific, Waltham, MA, USA) supplemented with 10% fetal bovine serum (FBS, Thermo Fisher Scientific), 2.5 μg of amphotericin, and a mixture of penicillin, streptomycin, and neomycin (Thermo Fisher Scientific). The cells were maintained at 37 °C in a humidified atmosphere with 5% CO_2_/95% O_2_. Cells were grown in 75 cm^3^ flasks and the cell culture medium was changed every three days. When the cells reached confluence (80%) they were harvested with trypsin and quantified with a hemocytometer. Cells were placed in artificial cerebrospinal fluid (aCSF: NaCl 147 mM, KCl 2.7 mM; MgCl_2_ 0.85 mM) at 10,000 cells/μL.

### 4.3. Stereotaxic Surgery

All animals were anesthetized using xylazine (10 mg/kg) and ketamine (35 mg/kg) and transferred to a stereotaxic device (Stoelting, Wood Dale, IL, USA). The control group was injected with aCSF (5 μL) and the glioma group with aCSF containing 50,000 RG2 cells at a rate of 1 μL/min in the right hemisphere, according to the following coordinates: AP +1, L +2.5 and V −4.5. The day of surgery was considered as day zero and treatments were started at day three.

### 4.4. Treatments

Control and glioma animals were randomly assigned to either of two groups (*n* = 10 in each). One group received nicotine treatment (1 mg/kg) injected subcutaneously for seven days starting on day three, the other group received vehicle (PBS 0.1 M). At day ten after surgery, the animals were sacrificed, and the brain was obtained. Glioma was obtained in 85% of the population that received RG2 cells, only the right hemisphere was processed for RNA extraction or isolation of microsomes.

### 4.5. Total RNA Isolation

Immediately after the sacrifice, the right brain hemisphere and liver were dissected over ice and incubated at room temperature in Trizol (Invitrogen; 1 mL/100 mg tissue). The tissues were homogenized by sonication (Branson Sonifier 450, Danbury, CT, USA). Total RNA was precipitated with isopropanol, then washed with 70% and 50% ethanol. RNA was dissolved in water treated with diethyl pyro-carbonate (DEPC) and quantified with a spectrophotometer (nanoDrop, Thermo Fisher Scientific). RNA integrity was assessed by electrophoresis in denaturizing formaldehyde agarose gels (1.5%) with a 3-morpholinopropane-1-sulfonic acid (MOPS) buffer, the gels were stained with ethidium bromide (Gibco).

### 4.6. cDNA Synthesis

First strand cDNA was synthesized using 5 μg of total RNA with Avian Myeloblastosis Virus (AMV) Reverse Transcriptase (Invitrogen) using an Oligo (dT) primer (Thermo Fisher Scientific). The reaction was incubated at 65 °C for 5 min, 45 °C for 60 min and 85 °C for 5 min to inactivate the reverse transcriptase.

### 4.7. Multiplex PCR

First strand cDNA was used for multiplex PCR amplification of *Cyp2b1/2*, *18s* and *GAPDH*. All reactions were performed in a final volume of 50 μL using PCR buffer (1×; BioTecMol, Mexico City, Mexico), magnesium chloride (liver: 1.5 mM; brain: 4 mM; BioTecMol), dNTPs (liver: 200 μM; brain: 400 μM; Thermo Fisher Scientific), oligonucleotides for *Cyp2b1/2* (liver: 300 nM; brain: 600 nM), *Gapdh* (liver: 120 nM; brain: 200 nM) and *18s* (200 nM), Taq polymerase (2.5 U; BioTecMol) and 1 μg of cDNA for each sample. The mixture was heated to 94 °C for 5 min and the PCR was performed for 26 (liver) or 30 (brain) cycles: 94 °C for 45 s, 61°C for 45 seconds and 72 °C for 45 s; finally, 72 °C for 5 min and the reaction was cooled to 4 °C. The amplicons were analyzed by electrophoresis in 2.5% agarose gels, which were stained with 1 μg/mL ethidium bromide and visualized with a UV Transilluminator (BioDoc-It Imagen System UVP M-20, Upland, CA, USA). A ladder of 100 bp (0.24 μg; Invitrogen) was used to identify their size.

### 4.8. Cyp2b1/2 Restriction Enzyme Digestion Analysis

The PCR product (25 μL) was digested with 10 U *Taq*αI (New England Biolabs, Ipswich, MA, USA) to eliminate unspecific amplification of *Cyp2b2*. Digestion with *Taqα*I was performed in NE buffer 3 supplemented with BSA (28 μg) at 65 °C for 1 h. Heating to 80 °C for 20 minutes inactivated the enzyme. The luciferase gene was included as a control to ensure that *Ta*qαI was working properly. The products were resolved in 2% agarose gels, stained with ethidium bromide, visualized and documented using BioDoc-It Image System M 20 (UVP) [27]. Densitometry analysis was performed using Quantity One software (v.4.6.5, Bio-Rad, Hercules, CA, USA).

### 4.9. Western Blot

The right hemisphere was homogenized by sonication on ice for four cycles at 100% amplitude during 20 s in 6 mL KCl (150 mM) supplemented with a protease-inhibitor cocktail (Complete, Roche Holding AG, Basel, Switzerland). Microsomes were isolated as follows: the homogenates were centrifuged at 9000× *g* during 10 min at 4 °C and the supernatants were recovered. Supernatants were centrifuged at 105,000× *g* for 1h at 4 °C and re-suspended in cold PB (pH 7.4) supplemented with a protease-inhibitor cocktail (Complete, Roche). The pellet was re-suspended in PB with 0.25 M sacarose and centrifuged at 105,000× *g* for 1 h at 4 °C. The pellet was recovered and re-suspended in PB supplemented with protease inhibitors and 20% glycerol. The protein concentration was assessed with bicinconinic acid assays [28]. Microsomal proteins from the brain (30 μg) and liver (10 μg) were separated by 10% sodium dodecyl sulfate polyacrylamide gel electrophoresis and transferred onto polyvinylidene difluoride membranes (PVDF, Millipore, MA, USA). The membranes were blocked with 5% dry milk in PBS (0.1 M) for 30 min and incubated overnight at 4 °C with a primary antibody against CYP2B1 (1:1000, AbNova MAB3815, Taipei, Taiwan), followed by a horseradish peroxidase (HRP) conjugated secondary anti-mouse antibody (1:10,000, Santa Cruz Biotechnology, Dallas, TX, USA). Immunoreactivity was visualized using enhanced chemiluminescence. After transfer, gel was stained with Coomassie blue as previously reported by Lawrence [29]. Densitometric analysis was performed using Quantity One software.

### 4.10. CYP2B1 Immunodetection

Double labeling for CYP2B1 and glial fibrillary acidic protein (GFAP) was performed on slide-floating brain sections. The tissue samples were treated with 0.1% sodium citrate for 1 h at 4 °C, 3% Triton X-100 in PBS for 1 h, and blocked with 5% bovine serum albumin in PBS for 1 h before incubation with the primary antibodies. The sections were incubated at 4 °C for 24 h with monoclonal mouse anti-CYP2B1 antibody (1:100; AbNova MAB3815) and rabbit polyclonal anti-GFAP antibody (1:500; Chemicon AB5804) in PBS. The sections were rinsed three times in PBS for 10 min each and incubated in the dark with Alexa 488 (anti-rabbit IgG; 1:500, Thermo Fisher Scientific) and Alexa 555 (anti-mouse IgG; 1:1000, Invitrogen) secondary antibodies for 2 h. After washing, nuclei were stained in a 1 μg/mL 4′,6-diamidino-2-phenylindole-dihydrochloride (DAPI) solution (Boehringer Mannheim GmbH, Mannheim, Germany), and the sections were coverslipped with fluorescence mounting medium (DAKO, Santa Clara, CA, USA). Control sections were treated in the same way but without primary antibody. Blood vessels detection were performed using anti-von Willebrand Factor antibody (FITC) (ab8822, 1:200; Abcam, Cambridge, UK). Images were collected with a Zeiss fluorescence microscope.

### 4.11. CYP2B6 Protein Detection in Human Glioma Samples

Brain glioma biopsies were obtained from pediatric patients at the Neurosurgery Department of the Instituto Nacional de Pediatría in México City, between November 2016 and August 2017. The tumor was resected and submitted to the pathology service for routine diagnoses, a portion was preserved for the study. All participants provided their written informed consent (M-2-0-25; M-2-027). The research procedures in our study were approved by the Ethics Committee in Research (INP 32/2018). Glioma specimens were scored for tumor grade by the pathology service, sample characteristics are listed in Table 1. All glioma tumors had novo origin and did not receive chemotherapy before surgery, except sample 1, which was a recurrent glioma treated with vincristine and carboplatin as well as radiotherapy at 64 G. Due to the small size of the tissue, total protein was obtained as follows. The tissue was homogenized by sonication, four cycles at 100% amplitude during 20 s in 500 μL of cold PBS (0.1 M) supplemented with a protease-inhibitors cocktail (Complete, Roche) and 30% glycerol. Homogenates were centrifuged at 12,500 rpm during 15 min at 4 °C and supernatants recovered. Protein samples (100 μg) were separated by 10% SDS-PAGE, transferred onto PVDF membranes, blocked with Odyssey Blocking Buffer (PBS, LI-COR) for 1 h at room temperature and incubated overnight at 4 °C with a primary rabbit antibody against CYP2B6 (1:1000, AbNova AB140609) followed by a IRDye 800CW goat anti-rabbit antibody (1:10,000; LI-COR 926-32211). Immunoreactivity was visualized with the Odyssey Imaging Systems. Densitometric analysis was performed using Quantity One software. 

### 4.12. Statistical Analysis

The statistical analysis was performed using GraphPad Software. Differential expression of the *Cyp2b1* gene and protein in rat liver and brain samples was compared among healthy and glioma animals treated with vehicle or nicotine using two-way analysis of variance (ANOVA) and Bonferroni post-hoc tests. A *p*-value less than 0.05 was considered significant.

## Figures and Tables

**Table 1:** Characteristics of the pediatric glioma samples.

Code	Gender	Age	Pathology Diagnoses	Grade Tumor *
1	male	10	Pylocitic astrocytoma	I
2	female	5	Gemystocitic astrocytoma	II
3	male	12	Pylocitic astrocytoma	I
4	male	6	Diffuse glioma	III
5	female	7	Glioblastoma	IV
6	male	14	Anaplasic Astrocytoma	III
7	male	3	Pylocitic astrocytoma	I

* Classification according to the World Health Organization (WHO). Low grade: I–II, high grade: III–IV.

## Abbreviations

CPA	Cyclophosphamide
CNST	Central nervous system tumors
WHO	World Health Organization
CYP450	Cytochrome P450
CAA	Chloroacetaldehyde
GFAP	Glial fibrillary acidic protein
MOPS	3-Morpholinopropane-1-sulfonic acid
AMV	Avian myeloblastosis virus
DAPI	4′,6-Diamidino-2-fenilindol-dihydrochloride
